# The SAFE Pilot Trial—SAlvage Focal Irreversible Electroporation—For Recurrent Localized Prostate Cancer: Rationale and Study Protocol

**DOI:** 10.3389/fsurg.2022.900528

**Published:** 2022-06-07

**Authors:** Giancarlo Marra, Taimur T. Shah, Daniele D’Agate, Alessandro Marquis, Giorgio Calleris, Luca Lunelli, Claudia Filippini, Marco Oderda, Marco Gatti, Massimo Valerio, Rafael Sanchez-Salas, Alberto Bossi, Juan Gomez-Rivas, Francesca Conte, Desiree Deandreis, Olivier Cussenot, Umberto Ricardi, Paolo Gontero

**Affiliations:** ^1^ Department of Surgical Sciences and Urology Clinic, University of Turin and Città della Salute e della Scienza, Turin, Italy; ^2^ Department of Urology and Clinical Research Group on Predictive Onco-Urology, APHP, Sorbonne University Paris, Paris, France; ^3^ Department of Urology, Imperial College, London, United Kingdom; ^4^ Department of Surgical Sciences and Radiology Clinic, University of Turin and Città della Salute e della Scienza, Turin, Italy; ^5^ Department of Urology, Centre Hospitalier-Universitaire Vaudois, CHUV, Lausanne, Switzerland; ^6^ Department of Urology, McGill University, Montreal, Canada; ^7^ Department of Radiotherapy, Institut Gustave Roussy, Villejuif, France; ^8^ Department of Urology, Hospital Clínico San Carlos, Madrid, Spain; ^9^ Department of Nuclear Medicine, University of Turin and Città della Salute e della Scienza, Turin, Italy; ^10^ Division of Radiotherapy and Department of Oncology, University of Turin and Città della Salute e della Scienza, Turin, Italy

**Keywords:** prostate cancer, biochemical recurrence (BCR), PSMA-PET/CT, irreversible electroporation (IRE), focal treatment

## Abstract

**Introduction:**

Currently, the majority of prostate cancer (PCa) recurrences after non-surgical first-line treatment are managed with androgen-deprivation therapy (ADT). Salvage radical prostatectomy (sRP) is a curative alternative to ADT but yields significant morbidity. Preliminary evidence from focal salvage treatments shows similar oncological control but lower morbidity compared to sRP. Among available ablative focal energies, irreversible electroporation (IRE) is a treatment modality that proved promising, especially in treating apical lesions, where PCa most often recurs. Our aim is to test the safety of salvage IRE for recurrent PCa.

**Methods:**

We performed a single-arm pilot feasibility study (IDEAL stage 2a): SAFE, SAlvage Focal irreversible Electroporation for recurrent localized PCa. Twenty patients with biopsy-proven PCa recurrence after primary non-surgical (radiation or ablation) treatment were included. All men will undergo mpMRI ± targeted biopsies, pre-operative PSMA-PET staging before inclusion and sIRE. Outcomes will be evaluated through internationally validated questionnaires and morbidity scales. All men will undergo a control biopsy at one year.

**Results:**

Primary objectives were the evaluation of the safety of sIRE (and patients’ quality of life) after treatment. Secondary objectives were the evaluation of functional outcomes, namely, continence and erectile function changes and evaluation of short-term oncological efficacy.

**Conclusions:**

SAFE is the second pilot study to evaluate sIRE and the first one performed according to the most recent diagnostic and staging imaging standards. sIRE may provide a curative option for recurrent PCa together with lower comorbidities compared to sRP.

## Introduction

Prostate cancer (PCa) is the most frequent non-skin solid neoplasm in men (1). Approximately 80% of the 190,000 new cases diagnosed yearly in the United States are found at a stage localized to the prostate (1). These patients undergo surgery in the majority of cases, but one in four chooses non-surgical treatments including radiotherapy (RT) and brachytherapy (BT) (2). Overall, reported rates of disease recurrence after RT and/or BT range from 10% to 30% at 5 years up to 50%–60% at 10 years (3–5). If considering a middle way, this would translate into 15,000 PCa recurrences per year, making radio-recurrent PCa the fourth most commonest male genitourinary cancer (1, 6).

Radio-resistant disease natural history shows half of the men not developing metastases at 5 years if left untreated. The other half will develop systemic progression at a median of 3 years. Hence, a significant proportion remains with a relevant window for a definitive cure (7). Similar figures are confirmed by recent PET-PSMA imaging studies, as more than half of recurrences are localized to the prostatic bed only (8, 9).

However, more than 90% of patients indiscriminately undergo palliative androgen-deprivation therapy (ADT), losing the chance of a definitive cure and eventually developing a castration-resistant state at a median of 2–3 years after the start of ADT (7). Also, healthcare will be faced with significant costs (10, 11): patients with several ADT-related side effects and decreased quality of life (QoL) (12). This disturbing compromise does not come as a surprise if acknowledging the results of the historical salvage radical prostatectomy series (sRP); major complications were experienced in up to one in three men, and median blood loss was described as up to almost 2L and incontinence in up to 80% of cases (13).

Results of multicenter series involving our and other institutions, and including the robotic approach, show relevant improvement of sRP morbidity in the contemporary era. Nonetheless, this surgery remains challenging. Rectal injuries are now rare (<1%–2%), and strictures also diminished (<10%–20%). However, currently, one in ten and one in three men still experience high-grade and overall complications, respectively (14–16); despite almost 60% of men preserving their pre-operative continence status, up to one in four still has severe (>3 pads/day) incontinence.

Similarly, oncological control is inferior to a first-line setting. Biochemical recurrence (BCR)-free survival is generally >60% at the end of a short- to intermediate-term follow-up; five-year progression-free (PFS) and cancer-specific survival (CSS) are around 50% and 90% at 5 years (17).

In recent years, there has also been an increasing interest in whole-gland and focal ablative strategies for primary PCa (18). Medium-term results of several energies have already proved promising (19). Nonetheless, on a longer follow-up, up to one in two men need some form of re-treatment due to PCa recurrence and/or persistence (20–23). Thus, recurrent PCa following non-surgical first-line treatment is further likely to grow in the near future.

Following partial ablation, sRP has proven comparable to a first-line setting rather than to surgery after radiotherapy, as complications are rare and continence is preserved in approximately 80%–90% of cases. However, erectile function preservation remains suboptimal and overall, there is still room for improvement (24, 25).

The rationale of focal treatments relies on treating the index cancer lesion, namely, the largest and more aggressive cancer focus, which likely drives PCa progression and metastatic spread, eventually leading to death (26). Contrarily, satellite non-significant lesions are unlikely to evolve and play a “clinically significant” role (19, 26). This concept has been criticized in a first-line setting and, in the presence of some contradictory evidence, likely requires further assessment (27).

Nonetheless, in a recurrent setting, PCa has been found in the same site of the original index lesion in 90% to up to 100% of the cases. First-line whole-gland treatment may definitively silence non-significant foci, while failure may be related to radio-resistant clones emerging within the index lesion (28–30). If further confirmed, this provides an evidence-based rationale for using focal strategies also in treating recurrences (30).

On the one hand, if proven to achieve adequate oncological control, focal salvage treatments (sFT) would dramatically reduce the rate of sRP-related complications. On the other hand, they would avoid ADT palliation and offer a curative option.

To date, results of more than 500 men receiving sFT have been detailed using brachytherapy, HIFU, or cryotherapy. The follow-up remains relatively short, with the majority of the series not reaching 5 years from treatment. Oncological control is promising, with 0%–20% developing metastasis at approximately 3 years and half of the men not having evidence of recurrence at 3–5 years. While results do not seem inferior to sRP, functional outcomes and complications are much improved: continence can be maintained in up to 90%; erectile function, when valid pre-operatively, generally has a slight decrease only; and complications, especially of high grade, are low, with strictures being detailed in less than 10% and most feared complications such as fistulas in less than 2% (31, 32).

Among new focal therapy energies, focal irreversible electroporation (fIRE) has proven promising due to its ability to cause direct cellular tissue damage through irreversible alterations of cell membrane permeability. Previous reports suggested only minimal damage to close structures surrounded by connective layers, including the urethral sphincter and neurovascular bundles, as IRE damages cells by disrupting the membrane equilibrium but not acellular connective tissues (33–35).

Furthermore, in a first-line setting, IRE has proven highly promising in the context of apical disease, with 90% failure-free survival at 3 years and only one in-field detailed recurrence in a series of 50 men with apical disease (36). This may represent an important advantage as, according to a recent analysis of sRP specimens, up to 90% of radio-recurrent PCa involves the prostate apex (37).

Nonetheless, the evidence in a salvage setting, where benefits in terms of morbidity and functional outcomes may be increased compared to a treatment-naïve scenario, is limited to a single-center series (38). Hence, we aim to perform a pilot single-center study to evaluate SAlvage Focal irreversible Electroporation for recurrent localized prostate cancer (SAFE) as the initial step to subsequently implement a larger phase II multicenter study.

## Methods and Analyses

### Study Objectives

#### Primary Objective

1. Evaluation of safety of focal irreversible electroporation for recurrent PCa and patients’ quality of life after treatment.

#### Secondary Objectives

1. Evaluation of functional outcomes of focal irreversible electroporation for radio-recurrent PCa, namely continence and erectile function changes;
2. Evaluation of short-term oncological efficacy.

#### Rationale

Our aim is to confirm evidence of the preliminary data from a single-center trial (38). If proving non-inferior results, we plan to proceed with a larger phase II study.

Our work complies with the IDEAL guidelines for evaluating surgical innovation in a phased manner. Our study represents stage 2a of these guidelines (prospective development study) (39, 40).

### Study Inclusion and Follow-Up

#### Pre-Operative Staging

**Figure 1:**
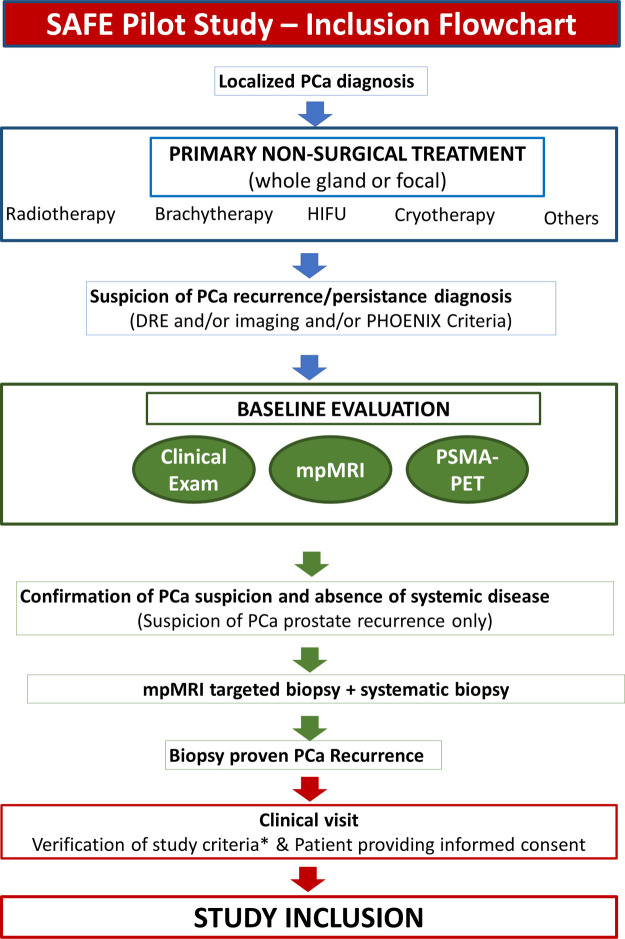
Flowchart illustrating the different steps from patient referral to study inclusion.

The process from patient referral to study inclusion is summarized in Figure 1. All men with clinical (DRE, mpMRI, PSMA-PET) and/or biochemical suspicion of recurrence (defined according to the PHOENIX Criteria—nadir PSA + 2 ng/ml) will undergo the following test to confirm the presence of PCa and stage the disease:

1. mpMRI and mpMRI visible lesion;
2. PSMA-PET;
3. mpMRI-targeted biopsies (in the case of a positive mpMRI) + systematic biopsies
  - including 14 cores transperineal biopsies—12 cores from the posterior zone and 2 cores from the anterior prostate bilaterally),
  - using a software able to provide prostate cartography (needle trajectories recorded) to be used to guide treatment needle positioning in case of subsequent treatment; and
4. Two PSA determinations in 3 months before treatment.

#### Study Criteria

##### Inclusion Criteria

- Biopsy-proven recurrent PCa—defined as recurrent after primary treatment with curative intent—including radiation therapy, HIFU, cryotherapy, and other ablation techniques. Both recurrences after focal or whole-gland primary treatment will be included;
- Clinically localized disease at mpMRI and PSMA-PET showing no extra-prostatic distant spread (pelvic nodes or other sites);
- Any Gleason score;
- Life expectancy >10 years;
- PSA≤20 ng/ml;
- Apical disease will be included;
- PCa suitable for focal salvage ablation up to hockey stick ablation, defined as treatment on three on four prostate quadrants; and
- Multifocal recurrent PCa involving more than three prostate quadrants (not suitable for hockey stick ablation).

##### Exclusion Criteria

- Prior ADT alone will not be considered as a previous treatment (e.g., patients with PCa after a cycle of ADT will not be considered for the study as ADT does not have a curative intent);
- Clinical T-stage cT4 and cT3b with >1 cm seminal vesicle involvement (mpMRI);
- Less than 6 months from primary treatment (persistent PCa);
- ADT performed in the 12 months before the treatment of recurrence;
- Patient history of epilepsy or cardiac arrhythmia or cardiac pacemaker;
- Recent history of myocardial infarction;
- Cardiac pacemaker;
- Active urinary tract and or other site infections;
- Ablation of lesions in the vicinity of implanted electronic devices or implanted devices with metal parts;
- Contraindications to performing mpMRI and/or PSMA-PET;
- Patients <18 years old; and
- Patients not providing written informed consent.

### Procedure

Salvage IRE will be performed using the Nanoknife system (Angiodynamics, Queensbury, NY, USA).

Patients will be placed in the lithotomy position after general anaesthesia—I.V. muscle paralysis and single-shot antibiotic prophylaxis. An indwelling urethral catheter will be placed.

**Figure 2:**
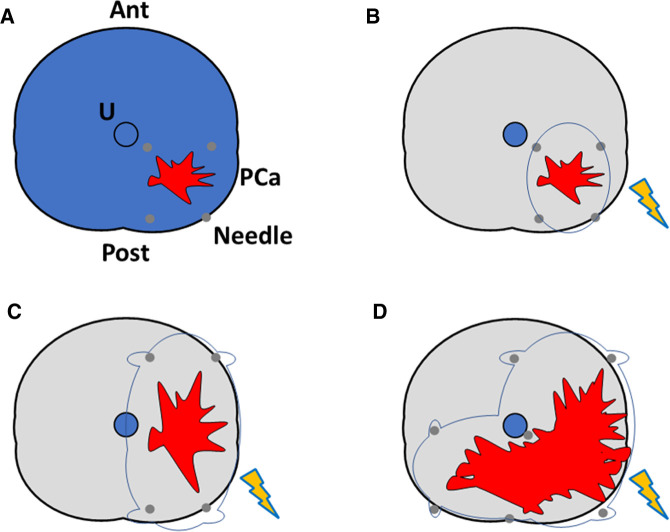
Different axial representations of salvage irreversible electroporation ablation schemes used in the SAFE study. Ant, anterior prostate; U, urethra; PCa, prostate cancer focus (red); Needle, irreversible electroporation needle (gray); ablation zone is displayed in orange. (**A**) Overall prostate view; (**B**) focal ablation; (**C**) hemi-ablation; (**D**) hockey stick ablation. Quadrant ablation will also be performed (not shown in the image).

A prostate biopsy using a co-axial needle will be taken at the site of needle placement before the procedure. The needle electrodes (19-gauge) will be placed through the perineum using TRUS guidance with a 5-mm brachytherapy template grid. A safety margin of 10 mm will be applied as previously described (38) based on biopsy and MRI evaluation. No Denonvilliers hydrodissection will be performed. The number of electrodes and active tip length depend on the required ablation size based on the recurrent PCa volume. The ablation template will also depend on lesion size, ranging from focal ablation to up to hockey stick ablation (Figure 2). A peri-procedural biopsy at the center of the needle placement will be performed before treatment delivery. *N* = 2 cores will be taken in the case of hockey stick ablation. The pulse features will be changed after a test using 20 test pulses to reach the required current for the ablative effect of IRE (20–40 A between each electrode pair). The remaining 80 treatment pulses will be then administered.

Patient discharge will be attempted on day 1 after catheter removal.

### Outcomes and Outcomes Measures

**Table:** 

	Outcome	Measure(s)
Primary outcomes—Timeline—12 months		
1.	Safety	National Cancer Institute Common Terminology Criteria for Adverse Events (CTCAE version 5.0) Clavien-Dindo
2.	Quality of life	Expanded Prostate Cancer Index Composite (EPIC) with specific urinary, sexual and bowel domains
Secondary outcomes—Timeline—12 months		
1.	Oncological control	Negative Prostate Biopsy
2.	Urinary function	IPSS and continence (pads/day)
3.	Sexual function	IIEF5 Ability to achieve penetrations (with/without PDE-5-I) Ejaculatory Function—Male Sexual Health Questionnaire Short Form

### Follow-Up

**Figure 3:**
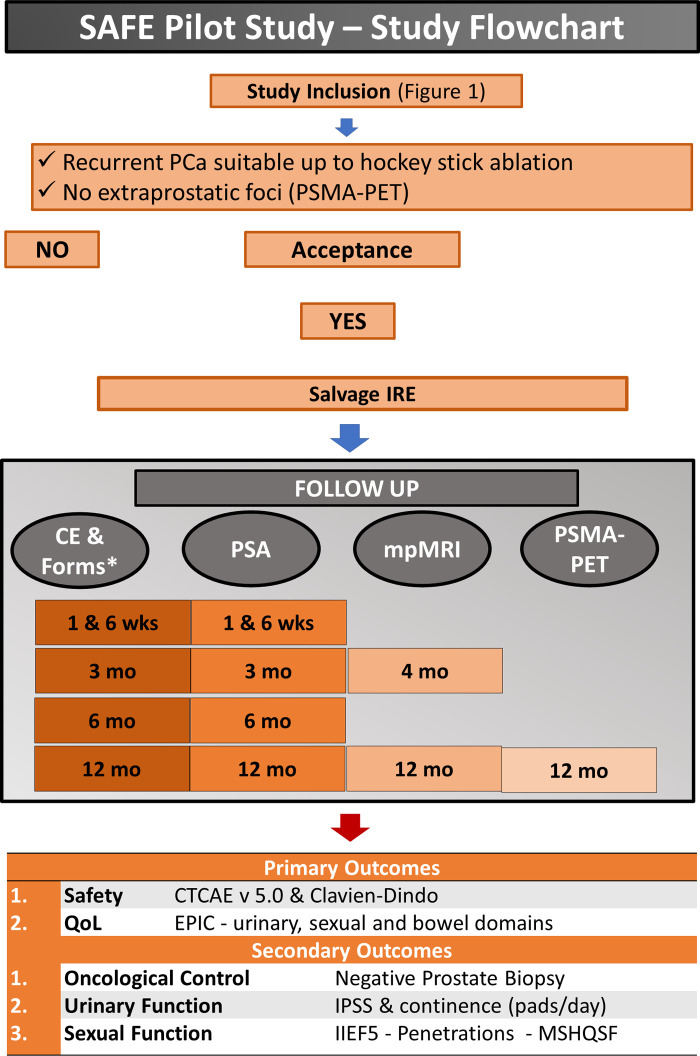
Flowchart illustrating the different steps of the study after patient inclusion. *=Clinical examination and questionnaires; at 1 week, a phone interview will be performed instead of a clinical visit; PCa, prostate cancer; IRE, irreversible electroporation; TCAE v 5.0, National Cancer Institute Common Terminology Criteria for Adverse Events; QoL, quality of life; EPIC, Expanded Prostate Cancer Index Composite—with specific urinary, sexual and bowel domains; IPSS, International Prostate Symptoms Score; IIEF-5, International Index of Erectile Function version 5.

The study flowchart from inclusion to follow-up is summarized in Figure 3. All study measures will be performed

- at 1 week (phone interview) and
- data manager for questionnaire plus clinical visit (6 weeks, 3 months, 6 months, and 12 months).

### Additional Oncological Assessment

- PSA at 1 week, 6 weeks, and 3, 6, and 12 months.

As per the recommended standards according to clinical practice and the latest evidence to maximize oncological results and PCa assessment and detection:

- mpMRI at 4 months (29, 38, 41, 42) and
- mpMRI (29, 38, 41, 42), PSMA-PET (43), and prostate biopsy (14-core systematic biopsy in the case of negative mpMRI) at 12 months—earlier in the case of PSA rise.

### Statistical Analysis

Our work corresponds to a pilot development study (stage IDEAL 2a).

As the primary objective of the study is to determine the safety profile of fsIRE, the sample size was calculated on the basis of complications. Previous work on fsIRE detailed two patients having CTCAE grade 2 adverse events (11%), five patients having grade 1 adverse events (27%), and no major complications (0%) (38). More recent sRP series detailed overall complications in approximately 30% and high-grade complications in 10% (44).

Considering an expected proportion of overall complications of 30%, the ±95% CIs around such proportions with *n* = 10, *n* = 20, and *n* = 25 would be at 62.6%, 45.6%, and 40.9%, respectively.

We have therefore chosen to set the sample size at *n* = 20, as there is significantly increased precision from *n* = 10 (Δ = 17.0%), but little improvement is achieved if more patients are included (Δ = 4.6%).

### Analysis Plan

The study is supposed to start in February 2022 and finish recruitment in August 2023 (18 months). Preliminary evaluation of the safety outcome will be evaluated at 6 months and 12 months. All primary and secondary outcomes will also be assessed on the first 10 patients in a 12-month follow-up.

## Discussion

### Summary and Strengths

SAFE is the second pilot study to evaluate safety and short-term oncological control of sIRE prospectively. Previous studies on focal salvage treatment did not include systematic staging and/or more recent diagnostic modalities (11, 31). In our study, all men will undergo mpMRI and, more importantly, PSMA-PET, which recently proved superior to conventional imaging by level 1 evidence in a staging setting (45). The use of pre-procedural PSMA-PET may help in excluding men with micro-metastases at the time of recurrence, potentially improving the overall treatment success rate and decreasing the rate of treatment with an unproven benefit (micro-metastatic patients). Also, the performance of mpMRI-targeted biopsies through elastic fusion software with the possibility of storing needle trajectories and outcomes will allow accurate treatment planning and electroporation needle positioning (46, 47).

The use of mpMRI targeted biopsies in a radio-recurrent setting is supported by the recent preliminary results of the FORECAST study, showing good accuracy of targeted compared to transperineal template mapping biopsies (48). Furthermore, we will add two cores to our previously published biopsy protocol (49), including systematic anterior zone sampling. In the absence of mpMRI and PSMA-PET suspicion and considering the generally low volume of prostate glands that previously received non-surgical treatment, this should allow to safely reduce the ablation zone, avoiding the anterior gland and, potentially, reducing morbidity (50, 51).

Finally, previous suggestions by experts agree with an “à la carte” approach, favoring the use of different energies depending on cancer features and location to maximize ablation efficacy (52–54). In this context, sIRE may potentially allow an overall optimal disease control in the posterior gland, in the anterior gland as the needles are positioned through perineal access (54), and in the apical prostate segments, where radio-recurrent disease frequently lies (37), as suggested by first-line fIRE series (36, 55, 56). If SAFE confirms safety and oncological control of other ablative energies and the FIRE trial, this will justify carrying out a larger trial looking at longer-term results of fsIRE in the context of a multicenter collaboration.

### Limitations

There are some relevant study limitations.

First, SAFE is a pilot trial. By definition, it aims to provide sufficient evidence favoring larger trials; however, it will not provide definitive evidence per se.

Second, the 12-month follow-up is sufficient to cover eventual toxicity. Nonetheless, it is too short to accurately record an estimate of stronger oncological endpoints such as metastatic progression, as this tends to occur at a median of 2–3 years from recurrence (7).

Third, we did not contemplate randomization. Although this has been recently proven feasible in a first-line setting (57), randomization may be even more difficult in a salvage context where one option, i.e., radical treatment, has potentially much higher morbidities and another, i.e., ADT, does not offer a curative chance. Nonetheless, we are aiming to prospectively collect data for men eventually preferring other treatment modalities/refusing sIRE off trial. These results may help in planning future sample size calculations in the case of randomized and/or non-randomized prospective cohort studies.

## Conclusions

Recurrence of PCa after non-surgical first-line treatment is not infrequent and likely to increase in absolute numbers. Currently, the majority of men undergo palliative ADT without being offered a curative chance. The main alternative is constituted by sRP, which, however, yields significant morbidities. In this setting, focal salvage treatment may potentially allow the same oncological control of radical treatment while significantly reducing its morbidity. Among ablative energies, sIRE may be advantageous as it has promising PCa control at the apex, where recurrent PCa is more frequently located. SAFE would be the second pilot study to evaluate sIRE and the first one performed according to the most recent diagnostic and staging imaging standards.
